# Impact of pulmonary artery capacitance index on early outcomes following paediatric heart transplantation

**DOI:** 10.1017/S1047951125100747

**Published:** 2025-07

**Authors:** Angela M. Monafo, William A. Harris, Carolyn L. Taylor, Minoo N. Kavarana, Jason R. Buckley, Andrew J. Savage, Anthony M. Hlavacek, Varsha M. Bandisode, John F. Rhodes, John M. Costello, Shahryar M. Chowdhury

**Affiliations:** 1 Division of Pediatric Cardiology, Medical University of South Carolina, Charleston, SC, USA; 2 Department of Pediatrics, Medical University of South Caroline, Charleston, SC, USA; 3 Section of Pediatric Cardiothoracic Surgery, Medical University of South Carolina, Charleston, SC, USA; 4 Cardiology, Medical University of South Carolina Shawn Jenkins Children’s Hospital, Charleston, SC, USA

**Keywords:** Indexed pulmonary artery capacitance, pulmonary vascular resistance, paediatric heart transplant, right ventricular failure

## Abstract

**Background:**

Pulmonary artery capacitance is a relatively novel measurement associated with adverse outcomes in pulmonary arterial hypertension. We sought to determine if preoperative indexed pulmonary artery capacitance was related to outcomes in paediatric heart transplant recipients, describe the changes in indexed pulmonary artery capacitance after transplantation, and compare its discriminatory ability to predict outcomes as compared to conventional predictors.

**Methods:**

This was a retrospective study of paediatric patients who underwent heart transplant at our centre from July 2014 to May 2022. Variables from preoperative and postoperative clinical, catheterisation, and echo evaluations were recorded. The primary composite outcome measure included postoperative mortality, postoperative length of stay in the top quartile, and/or evidence of end organ dysfunction.

**Results:**

Of the 23 patients included in the analysis, 11 met the composite outcome. There was no statistical difference between indexed pulmonary artery capacitance values in patients who met the composite outcome [1.8 ml/mmHg/m^2^ (interquartile 0.8, 2.4)] and those who did not [1.4 (interquartile 0.9, 1.7)], *p* = 0.17. There were no significant signs of post-operative right heart failure in either group. There was no significant difference between pre-transplant and post-transplant indexed pulmonary artery capacitance or indexed pulmonary vascular resistance.

**Conclusions:**

Preoperative pulmonary artery capacitance was not associated with our composite outcome in paediatric heart transplant recipients. It did not appear to be additive to pulmonary vascular resistance in paediatric heart transplant patients. Pulmonary vascular disease did not appear to drive outcomes in this group.

## Introduction

Early right ventricular failure is an important cause of postoperative morbidity and mortality in paediatric heart transplantation. Previous studies have identified complex CHD, pre-transplant pulmonary vascular resistance, and pre-transplant ventilator support, among others, as potential predictors of acute right ventricular failure after transplant.^1,2^ Right ventricular failure occurs even with acceptable pulmonary vascular resistance values in pre-transplant evaluation, suggesting that there are other factors which may be integral to predicting right heart failure after transplant.^1^ Pulmonary artery capacitance is a relatively novel measurement that has been shown to be associated with adverse outcomes in pulmonary arterial hypertension in paediatric and adult populations and in advanced heart failure in adults.^3–5^ However, no studies have investigated pulmonary artery capacitance’s relationship to outcomes after paediatric heart transplantation.

Pulmonary artery capacitance is defined as right ventricle stroke volume divided by pulmonary artery pulse pressure and has been used as a marker of right ventricle afterload.^4^ Pulmonary artery capacitance can be indexed to body surface area and used in the paediatric population, where it has been shown to be a strong predictor of outcomes in pulmonary arterial hypertension. Pulmonary artery capacitance was also found to have better predictive value than indexed pulmonary vascular resistance.^4^ In prior studies, it has been used in the evaluation of pulmonary arterial hypertension associated with CHD and was found to be associated with decreased survival at lower values.^7,8^

Orthotropic heart transplant relies on integration between the recipient lungs and donor heart. The pulmonary artery capacitance is an innate value to that recipient pulmonary vascular tree related to vessel size and dispensability with the additive influence over time by the hemodynamic effects of heart failure physiology. Increased pulmonary artery pressures will decrease pulmonary arterial compliance, a measure of wall stiffness. With prolonged exposure to elevated pulmonary artery pressures, the elasticity of the wall decreases as the elastin/collagen ratio is altered by vascular remodelling.^6^ Children with worsening or long-standing heart failure symptoms may be predisposed to increased remodelling of the pulmonary vasculature over time, which would decrease their pulmonary artery capacitance. At the time of transplantation, the donor right ventricle would be responsible for meeting the capacitance of the recipient pulmonary vasculature as defined by the innate pulmonary vascular resistance and any diastolic dysfunction of the transplanted left ventricle. If the ultimate capacitance is too low, this may predispose the donor right ventricle to premature failure and worse outcomes.

## Aims

We hypothesised that pulmonary artery capacitance is a more sensitive predictor of adverse outcomes after paediatric heart transplantation than pulmonary vascular resistance. However, the natural history and clinical utility of pulmonary artery capacitance has not yet been investigated in this population. The aims of this study were to 1) determine the relationship between preoperative pulmonary artery capacitance and adverse outcome following heart transplantation in our population; 2) compare the discriminatory ability of pulmonary artery capacitance to identify patients at risk for poor post-transplant outcomes versus conventional preoperative predictors of post-transplant outcomes, such as pulmonary vascular resistance; and 3) describe the natural history of pulmonary artery capacitance in the peri-operative period after heart transplantation.

This was a single centre retrospective cohort study that identified patients who had undergone paediatric heart transplantation at the Medical University of South Carolina from July 1 2014 to May 30, 2022. Patients that did not have a pre-transplant cardiac catheterisation, and patients who were palliated to single ventricle physiology with non-pulsatile pulmonary arteries were excluded. The Institutional Review Board approved this study.

Data from the patients’ catheterizations were retrospectively collected. Pre-transplant catheterisation, 1-week post-transplant, and 1-month post-transplant catheterisations were analysed. The most recent catheterisation before transplant was deemed the “pre-transplant catheterisation. All patients on continuous inotropic infusion or ventricular assist device had catheterisation before transplant as is our institutional protocol. Pulmonary artery capacitance is calculated as indexed right ventricular stroke volume divided by pulmonary artery pulse pressure. Right ventricular stroke volume was estimated as pulmonary blood flow measured in the catheterisation laboratory by Fick estimation, divided by heart rate, and indexed to body surface area. Pulmonary artery pulse pressure was calculated as pulmonary artery systolic pressure minus pulmonary artery diastolic pressure. While there are no “normal” values for pulmonary artery capacitance in children, one study showed that cumulative event-free survival rate is significantly lower when pulmonary artery capacitance indexed to body surface area is < 0.85 mL/mm Hg/m^2^ in paediatric pulmonary hypertension.^4^

Other conventional measures from the preoperative catheterisation, echocardiogram, and clinical assessment of patient-specific risk factors were included. Invasive haemodynamic data prior to transplant, at 1 week and 1 month post-transplant was recorded. Echocardiographic data post-transplant was assessed 1–3 days after transplant and at the time of 1 week and 1 month catheterisation. Right ventricular failure markers included invasive right ventricular end diastolic pressure and mean right atrial pressure, echocardiographic subjective right ventricular function, tricuspid annular systolic plane excursion, tissue Doppler s velocity, tricuspid regurgitation grade and gradient, and right ventricular fractional area of change.

A composite outcome variable was developed that incorporated postoperative length of stay in the top quartile, vasoinotropic score > 20 at any time postoperatively, postoperative multiorgan failure (Cr > 200% baseline, need for dialysis, total bilirubin > 2, and postoperative mechanical ventilation > 72 hours), need for extracorporeal membrane oxygenation, and/or mortality.

### Statistical analyses

Descriptive statistics were generated for study patients. Continuous variables were reported via medians (interquartile ranges). Categorical variables were summarised by counts and percentages. Differences between groups were assessed using Mann–Whitney U tests or Chi square/Fisher’s Exact test as appropriate. The relationship of pulmonary artery capacitance versus the composite outcome measure was analysed utilising logistic regression analysis. Changes in pulmonary artery capacitance over time were determined using the Kruskal–Wallis test with Bonferroni correction for multiple comparisons. A *p*-value < 0.05 was considered statistically significant. Data were analysed using SPSS v. 27 (IBM, Andover, MA).

## Results

Of the 40 patients who underwent heart transplantation in the study period, 23 subjects had catheterisation data that allowed calculation of pulmonary artery capacitance. Eleven patients (48%) met criteria to have reached the composite outcome. Pre-transplant patient characteristics and clinical data are shown in Table 1. There was no statistical difference between pre-transplant pulmonary artery capacitance values in patients who met the composite outcome and those who did not. Patients who met the composite outcome were more likely to have a lower pulmonary vascular resistance (*p* = 0.02). Pre-transplant total bilirubin was higher in the group that met the composite outcome (*p* < 0.01). Pre-transplant pulmonary artery capacitance had no relationship to the composite outcome upon logistic regression analysis (*p* = 0.06). Pre-transplant pulmonary vascular resistance had no relationship to the composite outcome upon logistic regression analysis (*p* = 0.10).

**Table 1.** Pre-transplant characteristics of the patient groups

	Did not meet composite (n = 12)	Did meet composite (n = 11)	*p*-value
Age at transplant (years)	2.5 (1.1, 11.9)	6.0 (1.7, 15.7)	0.35
Weight (kg)	14.5 (8.7, 71.7)	17.5 (18.5, 55.9)	0.74
Weight percentile (%)	79 (20, 86)	10 (1, 84)	0.26
BSA (m^2^)	0.58 (0.40, 1.75)	0.67 (0.41, 1.59)	0.74
Male sex, n (%)	7 (58)	9 (82)	0.37
Black race, n (%)	7 (58)	5 (46)	0.68
Non-Hispanic ethnicity, n (%)	12 (100)	11 (100)	1.00
Diagnosis, n (%)			0.33
CHD	2 (17)	3 (27)	
Dilated cardiomyopathy	7 (58)	4 (36)	
Restrictive cardiomyopathy	0	3 (27)	
Myocarditis	2 (17)	1 (9)	
Other	1 (8)	0	
Mechanical support, n (%)	8 (67)	9 (82)	0.64
Type of mechanical support, n (%)			0.47
LVAD	1 (100)	7 (77.8)	
BiVAD	0	2 (22.2)	
ECMO	0	0	
Inotropic support, n (%)	10 (83)	8 (72)	0.64
Ventilator support at time of transplant, n (%)	0 (0)	2 (18)	0.22
Growth failure (<5%ile wt), n (%)	2 (17)	4 (36)	0.37
O_2_ saturation (%)	100 (97, 100)	98 (95, 100)	0.38
Total Bilirubin (mg/dL)	0.4 (0.2, 0.5)	0.7 (0.5, 0.9)	0.01
Creatinine (mg/dL)	0.5 (0.3, 0.7)	0.5 (0.3, 0.8)	0.49
Inpatient prior to transplant, n (%)	8 (67)	9 (82)	0.64
Time from catheterisation to transplant (days)	58 (17, 147)	155 (38, 275)	0.29
Qpi (L/min/m2)	3.3 (2.4, 4.2)	3.1 (2.4, 3.9)	0.88
Heart rate (bpm)	126 (107, 156)	114 (85, 139)	0.26
mRAP (mmHg)	8 (5, 12)	8 (6, 10)	0.93
RV EDP (mmHg)	8 (5, 12)	8 (6, 10)	0.74
RV SVi (mL/m^2^)	23 (18, 35)	34 (21, 38)	0.20
PASP (mmHg)	40 (31, 42)	33 (28, 42)	0.38
PADP (mmHg)	20 (12, 23)	19 (14, 24)	1.00
PAPP (mmHg)	21 (16, 26)	15 (12, 23)	0.19
mPAP (mmHg)	29 (20, 31)	28 (21, 29)	0.79
PCWP (mmHg)	13 (9, 18)	19 (14, 20)	0.05
CI (L/min/m^2^)	3.3 (2.6, 4.2)	3.2 (2.3, 4.0)	0.82
PVRi (Wood units)	4.2 (3.5, 4.6)	2.8 (2.2, 3.1)	0.02
PVRi>3 Wood units, n (%)	7 (64%)	1 (9%)	0.01
PVRi>6 Wood units, n (%)	1 (8%)	1 (9%)	0.95
PACi (mL/mmHg/m^2^)	1.4 (0.9, 1.7)	1.8 (0.8, 2.4)	0.17
PACi ≤ 0.85 mL/mmHg/m^2^, n (%)	2 (17%)	2 (18%)	0.92

Data are presented as median (interquartile range) or count (percent). BiVAD = biventricular assist device; BSA = body surface area; CI = cardiac index; ECMO = extracorporeal membrane oxygenation; EDP = end-diastolic pressure; LVAD = left ventricular assist device; mPAP = mean pulmonary artery pressure; mRAP = mean right atrial pressure; PACi = indexed pulmonary artery capacitance; PADP = pulmonary artery diastolic pressure; PAPP = pulmonary artery pulse pressure; PASP = pulmonary artery systolic pressure; PCWP = pulmonary capillary wedge pressure; PVRi = indexed pulmonary vascular resistance; Qpi = indexed pulmonary blood flow; RV = right ventricle; Svi = indexed stroke volume.

Comparison of postoperative outcomes and measures of right ventricular health are shown in Table 2. Of note, mortality was low, with no early patient deaths, and just one death which occurred for unknown reasons > 1 year post transplant. No patient required post-transplant extracorporeal membrane oxygenation support. Median length of stay of 16 days (interquartile range 11, 27) after transplant for the entire cohort. There were no differences in measures of right ventricle health between the group that reached the composite outcome and the group that did not.

**Table 2.** Operative and postoperative data comparison between groups

	Did not meet composite (*n* = 12)	Did meet composite (*n* = 11)	*p*-value
**Post-surgical data**			
Donor ischaemic time (min)	218 (199, 252)	240 (220, 259)	0.32
Mortality, n (%)	0 (0)	1 (9)	0.48
Post op LOS (days)	12 (10, 16)	27 (15, 35)	<0.01
Max VIS Score	13 (10, 14)	19 (13, 27)	0.04
Days on inotropes	9 (6, 20)	12 (6, 34)	0.53
Days on iNO	1 (1, 2)	3 (1, 4)	0.03
Days of ventilator support	2 (1, 2)	4 (2, 6)	<0.01
Days with chest tube in place	5 (4, 6)	6 (4, 8)	0.26
Peak total bilirubin (mg/dL)	1.2 (0.7, 1.9)	1.9 (1.1, 3.6)	0.07
Creatinine ratio, max to baseline	1.3 (1.2, 1.7)	1.7 (1.6, 2.5)	0.05
**RV health immediately post-transplant**			
Days from transplant to echo, n (%)	2 (1, 2)	2 (2, 3)	0.13
Echo qualitative RV function, n (%)			0.09
Normal or mildly depressed	10 (83)	5 (46)	
Moderately or severely depressed	2 (17)	4 (54)	
Echo TR severity, n (%)			0.16
None/mild	11 (92)	7 (64)	
Moderate or severe	1 (8)	4 (36)	
RV FAC%	0.34 (0.27, 0.39)	0.28 (0.20, 0.33)	0.15
TR gradient (mmHg)	18 (16, 23)	18 (14, 25)	0.88
TAPSE (cm)	0.6 (0.4, 0.8)	0.6 (0.4, 0.9)	0.76
TAPSE z-score	−7.7 (−8.9, −5.6)	−8.1 (−9.0, −6.3)	0.56
TV S’ (cm/s)	5 (4, 7)	5 (4, 6)	0.40
TV S’ z-score	−3.3 (−3.7, −2.6)	−3.3 (−4.4, −2.8)	0.57
LV EF (%)	59 (54, 74)	58 (44, 62)	0.26
**RV health one-week post-transplant**			
Echo qualitative RV function, n (%)			0.40
Normal or mildly depressed	12 (100)	10 (91)	
Moderately or severely depressed	0 (0)	1 (9)	
Echo TR severity, n (%)			0.56
None/mild	12 (100)	10 (91)	
Moderate or severe	0 (0)	1 (9)	
RV FAC%	0.36 (0.34, 0.45)	0.32 (0.28, 0.39)	0.10
TR gradient (mmHg)	23 (19, 25)	23 (17, 28)	0.87
TAPSE (cm)	0.7 (0.6, 1.1)	0.7 (0.5, 0.8)	0.30
TAPSE z-score	−6.4 (−7.0, −5.4)	−8.4 (−9.4, −5.8)	0.07
TV S’ (cm/s)	6.4 (4.5, 7.4)	7.2 (6.3, 9.0)	0.33
TV S’ z-score	−2.8 (−3.2, −2.3)	−2.7 (−3.0, −1.8)	1.00
LV EF (%)	64 (59, 71)	60 (57, 66)	0.24
mRAP (mmHg)	9 (6, 11)	8 (6, 10)	0.50
RV EDP (mmHg)	9 (6, 11)	8 (6, 10)	0.50
PASP (mmHg)	34 (30, 37)	32 (25, 40)	0.87
PADP (mmHg)	15 (12, 20)	14 (11, 17)	0.72
PAPP (mmHg)	17 (12, 22)	15 (14, 20)	0.22
mPAP (mmHg)	23 (21, 27)	21 (19, 26)	0.54
PCWP (mmHg)	13 (10, 14)	11 (10, 13)	0.25
CI (mL/min/m^2^)	2.6 (2.5, 3.2)	2.6 (2.2, 3.2)	0.97
PVRi (Wood units)	3.5 (2.5, 6.3)	3.4 (2.8, 5.2)	0.97
PACi (mL/mmHg/m^2^)	1.7 (1.2, 1.9)	2.0 (1.1, 2.3)	0.38
**RV health one-month post-transplant**			
Echo qualitative RV function, n (%)			1.00
Normal or mildly depressed	12 (100)	11 (100)	
Moderately or severely depressed	0 (0)	0 (0)	
Echo TR severity, n (%)			1.00
None/mild	12 (100)	11 (100)	
Moderate or severe	0 (0)	0 (0)	
RV FAC%	0.37 (0.35, 0.41)	0.39 (0.33, 0.42)	0.79
TR gradient (mmHg)	21 (19, 24)	19 (16, 29)	0.71
TAPSE (cm)	0.9 (0.7, 1.2)	0.7 (0.6, 1.0)	0.17
TAPSE z-score	−4.7 (-7.0, -3.8)	−6.9 (-8.5, -6.3)	0.13
TV S’ (cm/s)	6.1 (4.9, 7.7)	4.7 (4.0, 9.2)	0.33
TV S’ z-score	−2.7 (-3.0, -2.5)	−3.1 (-4.0, -1.9)	0.41
LV EF (%)	60 (55, 67)	59 (53, 63)	0.57
mRAP (mmHg)	8 (6, 15)	10 (8, 12)	0.65
RV EDP (mmHg)	8 (6, 14)	11 (8, 12)	0.52
PASP (mmHg)	35 (26, 40)	32 (28, 38)	0.85
PADP (mmHg)	15 (12, 20)	15 (12, 20)	0.85
PAPP (mmHg)	15 (13, 20)	16 (12, 18)	0.89
mPAP (mmHg)	22 (18, 29)	20 (20, 28)	0.85
PCWP (mmHg)	11 (10, 16)	12 (11, 14)	0.48
CI (mL/min/m^2^)	2.6 (1.9, 3.0)	2.7 (2.4, 2.9)	0.80
PVRi (Wood units)	4.3 (2.9, 6.4)	3.6 (2.6, 7.0)	0.81
PACi (mL/mmHg/m^2^)	1.6 (1.2, 1.7)	1.6 (1.4, 1.9)	0.24

Data are presented as median (interquartile range) or count (percent). CI = cardiac index; EDP = end-diastolic pressure; EF = ejection fraction; FAC = fractional area change; iNO = inhaled nitric oxide; LOS = length of stay; LV = left ventricle; mPAP = mean pulmonary artery pressure; mRAP = mean right atrial pressure; PACi = indexed pulmonary artery capacitance; PADP = pulmonary artery diastolic pressure; PAPP = pulmonary artery pulse pressure; PASP = pulmonary artery systolic pressure; PCWP = pulmonary capillary wedge pressure; PVRi = indexed pulmonary vascular resistance; Qpi = indexed pulmonary blood flow; RV = right ventricle; Svi = indexed stroke volume; TAPSE = tricuspid annular plane systolic excursion; TR = tricuspid regurgitation; TV = tricuspid valve; VIS = vaso-inotropic score.

**Figure 1.**
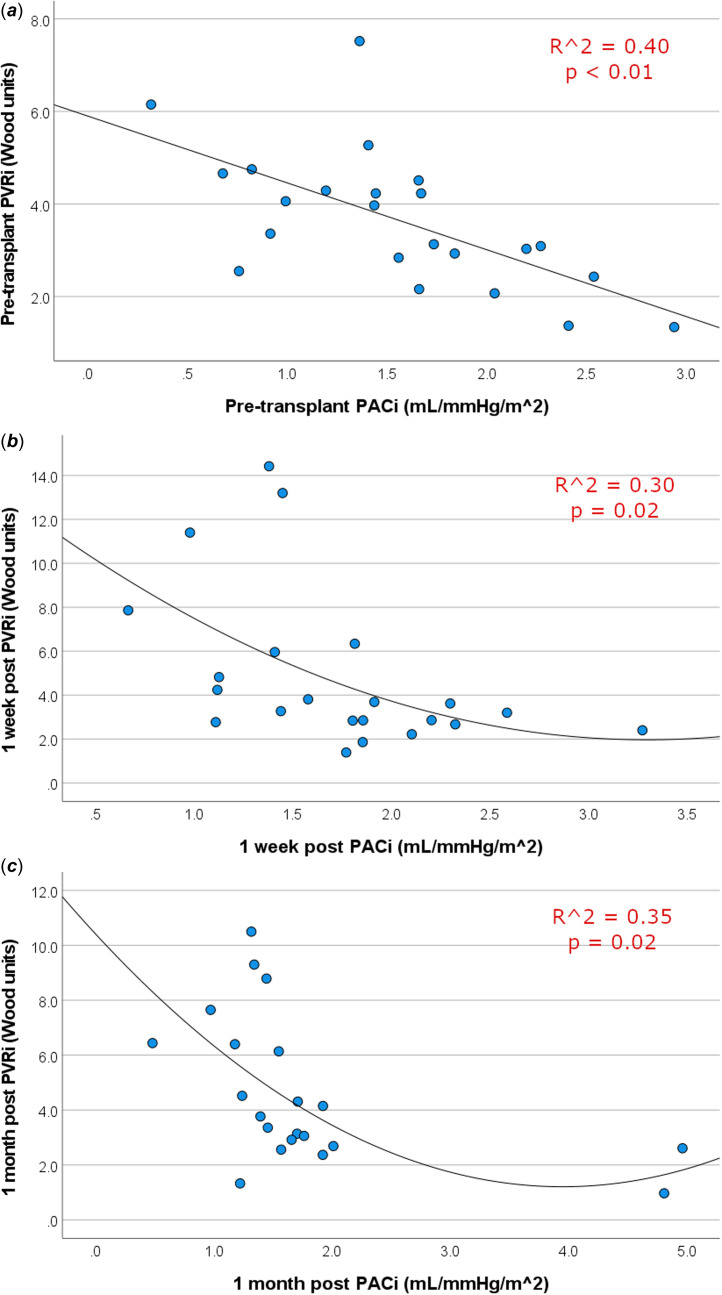
Scatterplots of PACi versus PVRi for data gathered pre-transplant (***a***) and at 1 week (***b***) and 1-month post-transplant (***c***).

Changes in invasive haemodynamic measurements over time are shown in Table 3. There were significant decreases in heart rate, pulmonary artery pressures, and pulmonary capillary wedge pressures before and after transplant at both 1 week and 1 month. Neither pulmonary vascular resistance nor pulmonary artery capacitance changed significantly between the pre- and post-transplant period. Pulmonary artery capacitance and pulmonary vascular resistance maintained significant associations with each other at the pre-transplant evaluation, as well as at 1 week and 1 month post transplantation (Figure 1).

**Table 3.** Changes in invasive haemodynamic data over time

Haemodynamics	Pre- transplant	1 Week	1 Month	*p* Value
CI (mL/min/m^2^)	3.3 (2.6, 4.0)	2.6 (2.4, 3.1)	2.7 (2.2, 2.9)	0.57
Qpi (mL/min/m^2^)	3.2 (2.4, 3.9)	2.6 (2.4, 3.1)	2.7 (2.2, 2.9)	0.26
HR (bpm)	120 (97, 139)	92 (86, 108)	96 (85, 119)	<0.01^*#^
SVi (mL/m^2^)	28 (21, 37)	27 (22, 34)	25 (22, 31)	0.77
mRAP (mmHg)	8 (5, 12)	8 (6, 10)	9 (7, 13)	0.31
RV EDP (mmHg)	8 (5, 10)	8 (6, 10)	9 (8, 13)	0.15
PASP (mmHg)	40 (29, 43)	34 (27, 38)	32 (28, 40)	0.02^*#^
PADP (mmHg)	19 (14, 24)	15 (12, 20)	15 (12, 20)	0.05*
PAPP (mmHg)	18 (14, 24)	16 (14, 20)	16 (13, 20)	0.92
mPAP (mmHg)	28 (21, 30)	22 (20, 26)	22 (20, 28)	0.03^*#^
PCWP (mmHg)	17 (10, 20)	12 (10, 14)	12 (10, 14)	<0.01^*#^
CI (mL/min/m^2^)	3.2 (2.6, 4.1)	2.7 (2.4, 3.2)	2.7 (2.5, 2.9)	0.57
PVRi (Wood units)	3.4 (2.6, 4.5)	3.4 (2.8, 6.1)	3.8 (2.7, 6.4)	0.95
PACi (mL/mmHg/m^2^)	1.6 (1.0, 2.0)	1.8 (1.1, 1.8)	1.6 (1.3, 1.8)	0.87

Data reported as median (interquartile range). Paired Kruskal–Wallis tests with multiple comparisons with Bonferroni corrections tests comparing haemodynamic data pre- and post-transplant at one week and one month. * = *p* < 0.05 for pre versus 1 week, # = *p* < 0.05 for pre versus 30 day, ^ = *p* < 0.05 for 1 week versus 1 month. CI = cardiac index; EDP = end-diastolic pressure; EF = ejection fraction; mPAP = mean pulmonary artery pressure; mRAP = mean right atrial pressure; PACi = indexed pulmonary artery capacitance; PADP = pulmonary artery diastolic pressure; PAPP = pulmonary artery pulse pressure; PASP = pulmonary artery systolic pressure; PCWP = pulmonary capillary wedge pressure; PVRi = indexed pulmonary vascular resistance; Qpi = indexed pulmonary blood flow, RV = right ventricle; Svi = indexed stroke volume.

## Discussion

The main finding of this study was that neither pulmonary artery capacitance nor pulmonary vascular resistance was predictive of outcomes in this sample of paediatric heart transplant recipients. Pulmonary artery capacitance and pulmonary vascular resistance remained unchanged through the early post-transplant period. Pulmonary vascular disease and right ventricular health were not drivers of patient outcomes in this study.

Prior studies have shown significant relationships between both pulmonary artery capacitance and pulmonary vascular resistance and suboptimal outcomes in cardiac transplant patients.^1,2,6^ These relationships were not found in the current study. We suspect this is due to the strict selection process utilised at our centre when identifying patients who are candidates to undergo cardiac transplantation, leaving very few patients with pulmonary vascular disease undergoing transplantation. For example, only four patients in the current study had pulmonary artery capacitance ≤ 0.85 ml/mmHg/m^2^ and only two patients and pulmonary vascular resistance greater than 6 Wood units. This suggests our cohort may not have exhibited enough pulmonary vascular disease for these markers to be a drivers of outcomes. It seems factors other than pulmonary vascular disease and right heart failure drove outcomes in this cohort. For example, the strongest preoperative measure associated with the composite outcome was total bilirubin, which is in line with prior published studies.^9,10^

Pulmonary artery capacitance and pulmonary vascular resistance are known to have a hyperbolic relationship. Scatterplots of our cohort comparing pulmonary artery capacitance to pulmonary vascular resistance are suggestive of this hyperbolic curve, reinforcing the relationship between these two measurements. This relationship results in a phenomenon where changes in pulmonary vascular resistance at high values is associated with very little change in pulmonary artery capacitance, while inversely at smaller pulmonary vascular resistance values, larger changes are seen in pulmonary artery capacitance with just slight change in pulmonary vascular resistance.^6^ In theory, this relationship may be beneficial when distinguishing between patients at risk for adverse outcomes in heart transplants, as pulmonary artery capacitance may be more discriminatory than pulmonary vascular resistance when the pulmonary vascular resistance is relatively low.^4^ However, our data suggest that pulmonary artery capacitance is not additive in paediatric heart transplant recipients with low pulmonary vascular resistance. Our results do not support its use in this setting.

In patients requiring heart transplantation, pulmonary hypertension often results from left-sided heart failure, or a “post-capillary” type hypertension. However, there is also a true risk of “mixed” pulmonary hypertension due to ongoing and additive structural changes to the pulmonary vasculature.^11,12^ Lim/Howell et al showed that left ventricular unloading with left ventricular assist device improved symptoms and clinical measures of mixed pulmonary hypertension. Cardiac transplantation should also result in analogous benefits to the pulmonary vasculature and improve mixed pulmonary hypertension over time.^13^ Dupont et al demonstrated how pulmonary artery capacitance can be improved over time with medical therapy for heart failure, suggesting that pulmonary artery capacitance is not a fixed variable and can be modified under a change in haemodynamic loading conditions. Our study did not show a difference in pulmonary artery capacitance or pulmonary vascular resistance between measurements taken prior to and post transplantation, nor differences in the value between the 1-week and 1-month post-transplant evaluation. There may be twofold reasons for this. The first is that 30 days may not be enough time to see a significant improvement in the pulmonary artery capacitance after heart transplantation. This may be compounded by the second reason, which is that at our centre pulmonary vascular resistance and diastolic dysfunction are treated aggressively, with high number of patients on IV milrinone and mechanical support prior to transplantation. These factors will decrease pulmonary vascular resistance, pulmonary capillary wedge pressure, and pulmonary artery pulse pressure, thus increasing pulmonary artery capacitance. Ultimately, this may result in a near optimised pulmonary artery capacitance even before transplant. However, given that there were significant changes in pre transplant and post-transplant measurements of pulmonary capillary wedge pressure and mean PA pressure, this suggests that more time may simply be required before there is a reflection in the pulmonary artery capacitance.

### Limitations

This study was limited by the sample size as well as its retrospective and single centre design. It is possible we failed to detect important predictors of paediatric transplant outcomes due to this limitation. Additionally, its utility is limited by the inability to estimate pulmonary artery capacitance in single ventricle patients with current formulas, particularly since failing Fontan patients are increasingly presenting for transplant consideration. The patient population in this study displayed little pulmonary vascular disease and right heart failure, making the generalizability of these results poor in such a patient population. It is possible that preoperative pulmonary artery capacitance is useful to predict heart transplant outcomes in a higher-risk cohort undergoing the procedure with more evidence of pulmonary vascular disease.

## Conclusions

Preoperative pulmonary artery capacitance was not associated with our composite outcome in paediatric heart transplant recipients. It did not appear to be additive to pulmonary vascular resistance in paediatric heart transplant patients. Pulmonary vascular disease did not appear to drive outcomes in this group. Pulmonary artery capacitance and pulmonary vascular resistance have an inverse relationship and do not change significantly in the first month after transplantation. Future studies should assess the utility of pulmonary artery capacitance in paediatric heart transplant recipients, utilising a larger, multi-centre cohort.
